# Tick- and fly-borne bacteria in ungulates: the prevalence of *Anaplasma phagocytophilum*, haemoplasmas and rickettsiae in water buffalo and deer species in Central Europe, Hungary

**DOI:** 10.1186/s12917-018-1403-6

**Published:** 2018-03-20

**Authors:** Sándor Hornok, László Sugár, Isabel G. Fernández de Mera, José de la Fuente, Gábor Horváth, Tibor Kovács, Attila Micsutka, Enikő Gönczi, Barbara Flaisz, Nóra Takács, Róbert Farkas, Marina L. Meli, Regina Hofmann-Lehmann

**Affiliations:** 1 Department of Parasitology and Zoology, University of Veterinary Medicine, Budapest, Hungary; 2 Department of Game Management and Ethology, Faculty of Agricultural and Environmental Sciences, University of Kaposvár, Kaposvár, Hungary; 3 SaBio. Instituto de Investigación en Recursos Cinegéticos (IREC-CSIC-UCLM-JCCLM), Ciudad Real, Spain; 4 Department of Veterinary Pathobiology, Center for Veterinary Health Sciences, Oklahoma State University, Stillwater, OK USA; 5 Veterinary Authority, Csurgó, Hungary; 6 Veterinary Authority, Mórahalom, Hungary; 7 Food Chain Safety and Veterinary Authority, Pásztó, Hungary; 8 Clinical Laboratory and Center for Clinical Studies, Vetsuisse Faculty, University of Zurich, Zurich, Switzerland

**Keywords:** *Anaplasma phagocytophilum*, *Mycoplasma wenyonii*, ‘*Candidatus* Mycoplasma haemobos’, *Mycoplasma suis*, *Rickettsia helvetica*

## Abstract

**Background:**

Hunting constitutes an important industry in Europe. However, data on the prevalence of vector-borne bacteria in large game animal species are lacking from several countries. Blood or spleen samples (239 and 270, respectively) were taken from red, fallow and roe deer, as well as from water buffaloes, mouflons and wild boars in Hungary, followed by DNA extraction and molecular analyses for *Anaplasma phagocytophilum*, haemoplasmas and rickettsiae.

**Results:**

Based on blood samples, the prevalence rate of *A. phagocytophilum* infection was significantly higher in red deer (97.9%) than in fallow deer (72.7%) and roe deer (60%), and in all these compared to mouflons (6.3%). In addition, 39.2% of the spleen samples from wild boars were PCR positive for *A. phagocytophilum*, but none of the buffalos. Based on blood samples, the prevalence rates of both *Mycoplasma wenyonii* (Mw) and ‘*Candidatus* M. haemobos’ (CMh) infections were significantly higher in buffaloes (Mw: 91.2%; CMh: 73.3%) than in red deer (Mw: 64.6%; CMh: 45.8%), and in both of them compared to fallow deer (Mw: 30.3%; CMh: 9.1%) and roe deer (Mw: 20%; CMh: 1.5%). The prevalence of Mw and CMh infection significantly correlated with the body sizes of these hosts. Furthermore, Mw was significantly more prevalent than CMh in buffaloes, red and roe deer. *Mycoplasma ovis* was detected in mouflons, *M. suis* in wild boars, *R. helvetica* in one fallow deer and one mouflon, and an unidentified *Rickettsia* sp. in a fallow deer.

**Conclusions:**

Forest-dwelling game animal species were found to be important carriers of *A. phagocytophilum*. In contrast, animals grazing grassland (i.e. buffaloes) were less likely to get infected with this *Ixodes ricinus*-borne pathogen. Water buffaloes, deer species, mouflons and wild boars harbored haemoplasmas that may affect domestic ungulates. Evaluated animals with larger body size had significantly higher prevalence of infection with haemoplasmas compared to smaller deer species. The above host species rarely carried rickettsiae.

## Background

Large game animal species, as exemplified by red deer (*Cervus elaphus*), roe deer (*Capreolus capreolus*) and wild boars (*Sus scrofa*), have increasing populations in Europe [1–3]. Adding to their ecological and economical significance, molecular screening of pathogens they may harbor also deserved special attention during the past decades. Taxonomically, large game animal species are artiodactyls (Mammalia: Cetartiodactyla), therefore it is expectable that they share pathogens with domestic even-toed ungulates [4]. In this context, there are various means of connectivity between game and domestic animals. These include hunting and offal waste disposal, the growing interface between urban and natural habitats, as well as blood-sucking arthropod vectors that may transmit disease agents between wild-living ungulates and livestock. The latter can happen even when they are more distantly separated.

Among arthropods, hard ticks (Acari: Ixodidae) are regarded as the most important vectors in the temperate zone [5]. Large game animal species are the preferred hosts of several tick species (e.g. *Ixodes ricinus* [6]), which have veterinary-medical significance [7]. Therefore, acting as reservoirs, they also play a central role in the maintenance and transmission of tick-borne infections towards humans. In particular, cervids and wild boars are regarded as reservoirs of tick- and/or vector-borne zoonotic pathogens, exemplified by *Anaplasma phagocytophilum* [8], *Mycoplasma suis* [9] and *Rickettsia helvetica* [10]. Accordingly, it was shown that changes in wildlife (especially deer) populations can contribute to the spread tick-borne infections [11].

In such epidemiological scenarios, it is especially important to know the actual prevalence of vector-borne infections in wild-living ungulates, because these infections may pose a risk of spreading to livestock or humans. However, relevant data are lacking from most of Europe. In general, more data are available on *A. phagocytophilum*, but virtually none considering haemotropic *Mycoplasma* spp. (synonymously: haemoplasmas). In Hungary, red deer and roe deer were shown to be important hosts of adults and immature stages of *I. ricinus* [12], and several *I. ricinus*-borne pathogens have been reported, for which game animals are known to be reservoirs (e.g. *A. phagocytophilum* [13] and *R. helvetica* [14]). In addition, haemoplasmas infecting cattle, sheep and pigs were found to be present in Hungary [15–17], but the carrier status of buffaloes, wild ruminants and wild boars has not been evaluated.

Therefore, the aim of this study was to survey and to compare the prevalence rates of selected, important vector-borne pathogens (i.e. *A. phagocytophilum*, haemoplasmas and rickettsiae) among water buffaloes (*Bubalus bubalis*) and five large game species: the red deer (*Cervus elaphus*), the fallow deer (*Dama dama*), the roe deer (*Capreolus capreolus*), the mouflon (*Ovis orientalis*) and the wild boar (*Sus scrofa*) in Hungary. In this country *A. phagocytophilum* and rickettsiae were detected in ticks [13, 14], unlike bovine haemoplasmas [14], which were shown to be fly-borne [15]. Therefore, it was hypothesized that molecular screening of the above hosts might also reveal differences in the prevalence of these infections depending on factors in an eco-epidemiological context (i.e. if the relevant pathogen has predominantly tick-borne or fly-borne transmission).

## Methods

### Sample collection

Samples were collected from water buffalos in a grassland natural reserve at Mórahalom (46° 13′ 4.5″ N, 19° 53′ 1.3″ E), and from freshly hunted individuals of five large game species in an approx. 3000 km^2^ forested region of south-western Hungary (46°15′-46°50’ N, 17°-17°50′ E), between January 2013 and December, 2014. None of these animals received acaricide or insecticide treatment. From water buffalos, blood samples were drawn from the jugular vein into EDTA tubes. From game animals, blood or spleen samples were collected randomly, depending on conditions allowed by hunters. Blood samples of dead game animals were taken from the heart (using sterile needle and syringe) and transferred into EDTA tubes, whereas spleen samples were cut with sterile scalpel blades and put into plastic vials. Samples included in the study are the following: 60 blood samples of water buffaloes; 48 blood and 96 spleen samples of red deer; 33 blood and 85 spleen samples of fallow deer; 65 blood and 6 spleen samples of roe deer; 16 blood and 4 spleen samples of mouflons; 17 blood and 79 spleen samples of wild boars. All samples were frozen on the day of collection at − 20 °C until processing.

### DNA extraction and molecular analyses

The DNA was extracted individually, from 200 μl blood or approx. 10 mg of spleen with the QIAamp DNA Mini Kit (Qiagen, Hilden Germany) according to the manufacturer’s instructions, including extraction controls to monitor cross-contamination of samples. PCR methods used for screening in this study are summarized in Table 1. In particular, *A. phagocytophilum* was identified with a species-specific TaqMan real-time PCR; haemoplasmas were first screened with a SYBR Green real-time PCR, then identified with species-specific TaqMan real-time PCRs, also taking into account their host-specificity; *R. helvetica* was screened with a species-specific test, and other rickettsiae with TaqMan real-time PCR. All molecular methods have been published (Table 1), except the following. The *Mycoplasma suis* assay was modified from [18]. Briefly, the 25-μl PCR reaction comprised 12.5 μl of 2× qPCR Mastermix (Eurogentec, Seraing, Belgium), 900 nM of each primer, 250 nM of probe and 5 μl of DNA. Assays were performed using an ABI PRISM 7500Fast Sequence Detection System (Thermo Fisher Scientific Inc., Waltham, MA, USA). The assay was validated using a ten-fold dilution series of a synthetic DNA containing the target sequence (GeneArt String DNA, Thermo Fisher Scientific Inc).

**Table 1:** Technical data and references of molecular methods used in this study

Target pathogen categories (target gene)	Oligonucleotides (sequence 5′-3′)	Reference for original method (modified protocol)
*Anaplasma phagocytophilum* (msp2)	ApMSP2f (ATG GAA GGT AGT GTT GGT TAT GGT ATT) ApMSP2r (TTG GTC TTG AAG CGC TCG TA) ApMSP2p (TGG TGC CAG GGT TGA GCT TGA GAT TG-HEX)	([13]) [35]
haemoplasmas (16S rRNA)	Sybr_For (AGC AAT RCC ATG TGA ACG ATG AA) Sybr_Rev1 (TGG CAC ATA GTT TGC TGT CAC TT) Sybr_Rev2 (GCT GGC ACA TAG TTA GCT GTC ACT)	[36] ([37])
*Mycoplasma wenyonii*, *M. ovis* (16S rRNA)	MwenyoniiF (CCA CGT GAA CGA TGA AGG TCT T) MwenyoniiR (GGC ACA TAG TTA GCT GTC ACT TAT TCA A) Mweny_P (FAM-AGT ACC ATC AAG GCG CGC TCA TTT CCT AG-TAMRA)	[38]
‘*Candidatus* M. haemobos’ (16S rRNA)	Mwen_short.forw (CCA TGT GAA CGA TGA AGG TCT TT) Mwen_short.rev (AGT TTG CTG TCA CTT ATT CAT GAG GTA) Mwen_short.p (VIC-CTA TCA GTT RTT ATC CCT CAT AA-MGB)	[38]
*Mycoplasma suis* (16S rRNA)	RTsuisF (CCC TGA TTG TAC TAA TTG AAT AAG) RTsuisR (GCG AAC ACT TGT TAA GCA AG) MGBsuis2 (FAM- TGR ATA CAC AYT TCA G -MGBNFQ)	[18]
*Rickettsia helvetica* (23S rRNA)	Rickhelv.147f (TTT GAA GGA GAC ACG GAA CAC A) Rickhelv.211r (TCC GGT ACT CAA ATC CTC ACG TA) Rickhelv.170p (6FAM-AAC CGT AGC GTA CAC TTA-MGBNFQ)	[39]
other rickettsiae (gltA)	CS-F (TCG CAA ATG TTC ACG GTA CTT T) CS-R (TCG TGC ATT TCT TTC CAT TGT G) CS-P (FAM-TGC AAT AGC AAG AAC CGT AGG CTG GAT G-BHQ)	[39]

In addition, amplification and sequencing an approx. 850 bp fragment of the major surface protein 4 (*msp4*) gene of *A. phagocytophilum* was attempted from a representative number of samples (i.e. 4–5 samples with low Ct value per host species). This PCR was performed with the primers MSP4AP5 (5′-ATG AAT TAC AGA GAA TTG CTT GTA GG-. 3′) and MSP4AP3 (5′-TTA ATT GAA AGC AAA TCT TGC TCC TAT G-3′) as reported [19]. The sequences were aligned and compared to reference GenBank sequences by nucleotide BLASTn program (https://blast.ncbi.nlm.nih.gov). Phylogenetic analysis was performed with the Neighbor-Joining method and p-distance model. Sequences were submitted to GenBank (accession numbers: MF974848–60).

Each PCR was run with positive and negative controls (i.e. sequence-verified DNA extract of the relevant agent, and non-template reaction mixture, respectively). Positive controls were always PCR positive, whereas negative controls and extraction controls remained PCR negative.

### Data analyses

Exact confidence intervals (CI) of the prevalence rates were calculated at the 95% level. The ratios of positive and negative samples were compared by Fisher’s exact test. In the case of host species, of which at least 20 blood samples were available, Spearman rank correlation was used to test the association between the prevalence of haemoplasmas and host body weights. Typical body weight range (i.e. locally characteristic of each species) (Table 2) was assigned according to the local hunting bags, taking into account differences between male and female animals (provided by Prof. L. Sugár). Calculations were performed with both the minimum and maximum body weights. Differences were regarded significant if *P* < 0.05.

**Table 2:** Results of molecular analyses of DNA samples from water buffalos and large game animal species

Species (body weight range)^a^	Sample type	PCR positives/all tested (percentage)				
		*Anaplasma phagocytophilum*	haemotropic *Mycoplasma* sp.			*Rickettsia helvetica* [other rickettsiae]
			*M. wenyonii* [*M. ovis*]	*C. M. haemobos*	*M. suis*	
Water buffalo (300–500 kg)	blood	0/60	55/60 (91.2%)^c, B^	44/60 (73.3%)^c, A^	–	0/60
Red deer (100–200 kg)	blood	47/48 (97.9%)^c^	31/48 (64.6%)^b^	22/48 (45.8%)^b^	–	0/48
	spleen	95/96 (99%)	24/96 (25%)^B^	4/96 (4.2%)^A^	–	0/96
Fallow deer (50–100 kg)	blood	24/33 (72.7%)^b^	10/33 (30.3%)^a^	3/33 (9.1%)^a^	–	0/33
	spleen	81/85 (95.3%)	6/85 (7.1%)	10/85 (11.8%)	–	1/85 (1.2%) [1/85]
Roe deer (25 kg)	blood	39/65 (60%)^b^	13/65 (20%)^a, B^	1/65 (1.5%)^a, A^	–	0/65
	spleen	3/6 (50%)	3/6 (50%)	0/6	–	0/6
Mouflon	blood	1/16 (6.3%)^a^	[1/16 (6.3%)]	0/16	–	1/16 (6.3%)
	spleen	4/4 (100%)	[1/4 (25%)]	0/4	–	0/4
Wild boar	blood	6/17 (35.3%	–	–	8/17 (47.1%)	0/17
	spleen	31/79 (39.2%)	–	–	23/79 (29.1%)	0/79

^a^Estimated body weight range characteristic of the species (i.e. minimum to maximum acc. to females and males) based on local hunting bags (provided by Prof. L. Sugár)

Significance: (1) blood sample-based prevalences, which are significantly different between host species, are marked with different superscript lower case letter within columns, increasing from “a” to “c”; (2) the prevalence of *M. wenyonii* is marked with superscript capital letter “B”, if significantly higher than that of *C.* M. haemobos marked with superscript capital letter “A” in the same row; (3) arrows in circle with inverse color point to the significantly higher prevalence, when comparing the two kinds of sampled tissues (blood vs. spleen) for the same host and pathogen

## Results and discussion

### Anaplasma phagocytophilum

*Anaplasma phagocytophilum* was detected in all evaluated species, except buffaloes (Table 2). These buffaloes are kept in a grassland, which is an unsuitable habitat type for *I. ricinus* as a forest-dwelling tick species [20, 21]. This is the most likely explanation, why buffaloes did not have *A. phagocytophilum* infection, while its prevalence was high in game animal species hunted in forests (Table 2).

Concerning blood samples of game animals (Table 2), the prevalence rate of *A. phagocytophilum* infection was significantly higher in red deer (97.9%, CI: 88.9–100%) than in fallow deer (72.7%, CI: 54.5–86.7%) and roe deer (60%, CI: 47.1–72%) (*P* = 0.001), and in all these compared to mouflons (6.3%, CI: 0.2–30.2%) (*P* = 0.0001). These data confirm the reservoir role of cervids in the epidemiology of *A. phagocytophilum* as known from other studies (reviewed in [8]). An important underlying factor to fulfill this reservoir role is that wild ruminants are a preferred host of *I. ricinus*, the vector of *A. phagocytophilum*, as also reported in Hungary [12].

The DNA of *A. phagocytophilum* was detected in 39.2% (CI: 28.4–50.9%) of spleen samples and 35.3% (CI: 14.2–61.7%) of blood samples from wild boars (Table 2). The prevalence of *A. phagocytophilum* in wild boars was shown to be lower in other European countries [8, 22]. Wild boars (as contrasted to cervids) are hosts of the zoonotic variant of *A. phagocytophilum* [23, 24], therefore these preliminary results justify further investigations on the role of wild boars in the epidemiology of human granulocytic anaplasmosis in Hungary.

**Fig. 1:**
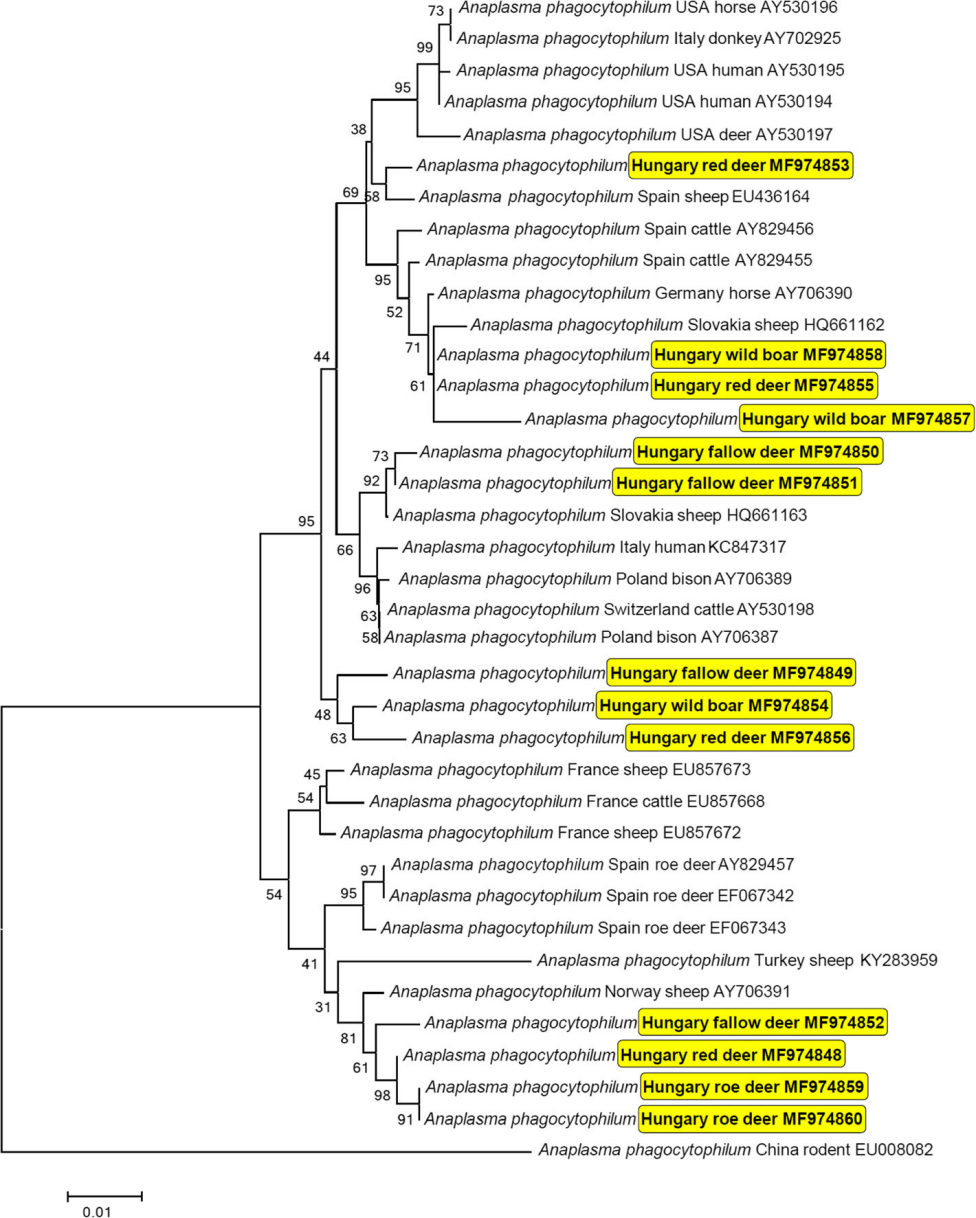
Neighbor-Joining phylogenetic tree based on the *msp4* gene of *Anaplasma phagocytophilum*, including sequences obtained in this study (indicated with yellow background) and representative sequences from GenBank. Before the accession number, the country and host of origin is shown. Branch lengths represent the number of substitutions per site inferred according to the scale shown

The *msp4* gene fragment of *A. phagocytophilum* was successfully sequenced from 13 samples (none from mouflons). The highest degree of sequence heterogeneity was observed among samples from red deer, which had up to 3.9% difference (i.e. 96.1% = 816/849 bp similarity). Phylogenetic analysis of *A. phagocytophilum msp4* sequences did not show clustering according to host species (Fig. 1). However, the phylogenetic tree reflected a geographical pattern, because sequences from different host species sampled in Hungary frequently grouped together (Fig. 1).

The DNA of *A. phagocytophilum* was significantly more frequently amplified from spleen than from blood samples of fallow deer and mouflons (*P* = 0.001). This phenomenon is similar to that reported in wild boars in Slovenia [25] and in various game animal species in Poland [26], most likely due to the density of *A. phagocytophilum*-infected granulocytes in the spleen compared to peripheral blood [24].

### Haemoplasmas

Haemotropic *Mycoplasma* spp. were detected in all host species evaluated in the study (Table 2). Based on blood samples (Table 2), the prevalence rates of both *Mycoplasma wenyonii* (Mw) and ‘*Candidatus* M. haemobos’ (CMh) infections were significantly higher in buffaloes (Mw: 91.2%, CI: 81.6–97.2%; CMh: 73.3%, CI: 60.3–83.9%) than in red deer (Mw: 64.6%, CI: 49.5–77.8%; CMh: 45.8%, CI: 31.4–60.8%) (*P* = 0.0007 and 0.005, respectively); and in red deer compared to fallow deer (Mw: 30.3%, CI: 15.6–48.7%; CMh: 9.1%, CI: 1.9–24.3%) (*P* = 0.003 and 0.0005, respectively) and roe deer (Mw: 20%, CI: 11.1–31.8%; CMh: 1.5%, CI: 0.1–8.3%) (*P* < 0.0001). In addition, Mw was significantly more prevalent than CMh in buffaloes (*P* = 0.015), in roe deer (*P* = 0.001) and (based on spleen samples) in red deer (*P* < 0.0001) (Table 2). These data prove that cervids and buffaloes are important carriers of haemotropic *Mycoplasma* spp. infecting cattle, to the best of our knowledge, observed for the first time in Europe.

Based on blood samples, there was a highly significant (*r* = 1) positive correlation between the body weights (i.e. body size) of buffaloes and deer species and the prevalence rates of Mw and CMh infections (Table 2). One possible background factor of this observation might be that larger animals (especially if living in an open area, as buffaloes), are more frequently exposed to the bites of blood-sucking vectors of Mw and CMh compared to smaller deer species. The most likely vectors of bovine haemoplasmas in Hungary are neither blood-sucking lice (absent from deer and the relevant buffalo herd: [27]) nor ticks [14], but blood-sucking flies [15], which are known to be competent mechanical transmitters of haemoplasmas [28]. Blood-sucking flies were shown to be attracted to their hosts according to body size-dependent stimuli, such as visibility and odor emission [29]. On the other hand, further factors (e.g. host susceptibility, environmental variables) might have also influenced these prevalence rates.

Interestingly, the DNA of bovine haemoplasmas was detected significantly more often in blood than in spleen samples: for Mw in red deer (*P* < 0.0001) and fallow deer (*P* = 0.002), and for CMh in red deer (*P* < 0.0001) (Table 2). This may reflect relatively recent infection in the majority of animals and/or the quick elimination of haemoplasma DNA in the spleen following phagocytosis. Relevant to this, splenic sequestration and phagocytosis of haemoplasmas has been reported [30].

*Mycoplasma ovis* was detected in mouflons (Table 2). To the best of the authors’ knowledge, there are no previous reports on the molecular identification of this haemoplasma in wild-living small ruminants in Europe.

*Mycoplasma suis* infection was demonstrated in wild boars (Table 2) at a significantly higher prevalence rate (based on blood samples: 47.1%, CI: 23–72.2%) than reported in Germany (i.e. 10%: [31]). This is the first evidence of *M. suis* infection in wild boars in Hungary. The importance of this finding is enhanced by the known zoonotic potential of *M. suis* [32] and recently reported emergence of wild boars in densely populated urban areas of Hungary [33].

### Rickettsiae

*Rickettsia helvetica* was detected only in one fallow deer and one mouflon (to the best of our knowledge, for the first time in these host species), and an unidentified *Rickettsia* sp. in a fallow deer (Table 2). Therefore, in contrast to the above categories of vector-borne pathogens, buffaloes and large game animal species do not appear to be important carriers of rickettsiae in the evaluated region. These low rates of infections are consistent with those in reports of *R. helvetica*-infected game animals in Central and Western Europe [10, 34].

## Conclusions

In the evaluated region, forest-dwelling cervids have different prevalence rates of infection with *A. phagocytophilum*, the highest observed in red deer. The prevalence of *A. phagocytophilum* was also high in wild boars compared to other countries. In contrast, this *I. ricinus*-borne pathogen was not detected in animals grazing grassland (i.e. buffaloes). Among haemoplasmas that may affect domestic ungulates, Mw/CMh occur in water buffaloes and deer species, whereas *M. ovis* in mouflons. Larger animals (especially if living in an open area, as buffaloes) were shown to have significantly higher prevalence rates of infection with bovine haemoplasmas. Wild boars had high prevalence of *M. suis* infection, what is important in light of its known zoonotic potential. *Rickettsia helvetica* was detected in fallow deer and mouflon. The rare occurrence of rickettsiae in the evaluated hosts is in line with data reported from other regions of Europe.

## Abbreviations

CI: exact confidence interval
CMh: *Candidatus* Mycoplasma haemobos
Mw: *Mycoplasma wenyonii*
